# Characterization of candidate genes involved in halotolerance using high-throughput omics in the halotolerant bacterium *Virgibacillus chiguensis*

**DOI:** 10.1371/journal.pone.0201346

**Published:** 2018-08-09

**Authors:** Yan-Huey Chen, Yuan-Tay Shyu, Shih-Shun Lin

**Affiliations:** 1 Department of Horticulture and Landscape Architecture, National Taiwan University, Taipei, Taiwan; 2 Institute of Biotechnology, National Taiwan University, Taipei, Taiwan; 3 Agricultural Biotechnology Research Center, Academia Sinica, Taipei, Taiwan; 4 Center of Biotechnology, National Taiwan University, Taipei, Taiwan; 5 National Center for High-Performance Computing, National Applied Research Laboratories, Hsinchu, Taiwan; Universite Paris-Sud, FRANCE

## Abstract

We previously used whole-genome sequencing and Tn5 transposon mutagenesis to identify 16 critical genes involved in the halotolerance of *Halomonas beimenensis*, a species in the phylum *Proteobacteria*. In this present study, we sought to determine if orthologous genes in another phylum are also critical for halotolerance. *Virgibacillus* spp. are halotolerant species that can survive in high-saline environments. Some *Virgibacillus* species are used in different aspects of food processing, compatible solute synthesis, proteinase production, and wastewater treatment. However, genomic information on *Virgibacillus chiguensis* is incomplete. We assembled a draft *V*. *chiguensis* strain NTU-102 genome based on high-throughput next-generation sequencing (NGS) and used transcriptomic profiling to examine the high-saline response in *V*. *chiguensis*. The *V*. *chiguensis* draft genome is approximately 4.09 Mbp long and contains 4,166 genes. The expression profiles of bacteria grown in 5% and 20% NaCl conditions and the corresponding Gene Ontology (GO) and clusters of orthologous groups (COG) categories were also analyzed in this study. We compared the expression levels of these 16 orthologs of halotolerance-related genes in *V*. *chiguensis* and *H*. *beimenensis*. Interestingly, the expression of 7 of the 16 genes, including *trkA2*, *smpB*, *nadA*, *mtnN2*, *rfbP*, *lon*, and *atpC*, was consistent with that in *H*. *beimenensis*, suggesting that these genes have conserved functions in different phyla. The omics data were helpful in exploring the mechanism of saline adaptation in *V*. *chiguensis*, and our results indicate that these 7 orthologs may serve as biomarkers for future screening of halotolerant species in the future.

## Introduction

The genus *Virgibacillus* belongs to the *Bacillaceae* family within the *Firmicutes* phylum [1]. The members of *Virgibacillus* have mostly been isolated from saline environments [1]. Thirty-nine species in the *Virgibacillus* genus have been validated and published in the NCBI database (NCBI database, 2017), whereas 381 other strains have non-validated species names. *Virgibacillus alimentarius* has been used for extracellular lipolytic enzyme purification [2], and *Virgibacillus pantothenticus* produces an extracellular thermostable serine alkaline protease [3].

Members of *Virgibacillus* can be found in different food products, such as fermented jeotgal, Dongcai, and fish sauce [4]. Like many halophilic and halotolerant bacteria, members of the *Virgibacillus* genus synthesize various types of compatible solutes. For instance, *Virgibacillus salexigens*, *V*. *marismortui*, *V*. *halodenitrificans*, and *V*. *pantothenticus* have been shown to produce ectoine, proline, glycine betaine, or glutamic acid in response to high salinity [5–9].

Currently, complete genomic sequences are available for 6 *Virgibacillus* species (NCBI database, 2017). For instance, the complete genome of *Virgibacillus halodenitrificans* PDB-F2, which is used for phenol degradation in wastewater treatment, has been analyzed [9]. Compatible solute synthesis and transport were further investigated by whole-genome sequencing [9]. Moreover, the production of an NaCl-activated extracellular proteinase was studied in *Virgibacillus* sp. SK37, which is popularly used in fish sauce production, and novel enzymes that can function in high-salt conditions were identified [10–12]. In addition, incomplete genomic contigs of *Virgibacillus chiguensis* strain CGMCC 1.6496 (FQXD01000000.1) were published on NCBI by the Joint Genome Institute. There are 63 contigs and 45 scaffolds for the CGMCC 1.6496 strain. The size of the CGMCC 1.6496 strain is 4.13 Mb with 3,858 annotated genes.

The genomic sequence of another halotolerant bacterium, *Halomonas beimenensis* NTU-111, which belongs to the phylum *Proteobacteria*, was obtained in our previous study and used to analyze its saline adaptation mechanism [13]. The *H*. *beimenensis* genome was sequenced using short and long reads of genomic sequence that were generated by next-generation sequencing (NGS) to assemble a complete genome [13]. Based on the transcriptomic profiles and Tn5 transposon mutagenesis, sixteen halotolerance-related genes were identified, and the possible molecular mechanisms of saline adaptation were examined [13]. Orthologs of *nqrA*, *trkA2*, *nadA*, and *gdhB* have significant biological functions in sodium efflux, potassium uptake, hydrogen ion transport for energy conversion, and compatible solute synthesis, respectively [13]. Other genes, such as *spoT*, *prkA*, *mtnN2*, *rsbV*, *lon*, *smpB*, *rfbC*, *rfbP*, *tatB*, *acrR1*, and *lacA*, function in cellular signaling, quorum sensing, transcription/translation, and cell motility, which have also been shown to be critical for promoting halotolerance [13]. Therefore, we examined whether orthologs of these 16 genes in a different phylum also play a role in halotolerance.

In this study, we isolated *Virgibacillus chiguensis* strain NTU-102 (hereafter referred to as *V*. *chiguensis*), which was collected from the Chigu saltern of Taiwan, and its entire genome was sequenced using NGS. Furthermore, *V*. *chiguensis* gene expression profiles under various salt conditions were analyzed by transcriptome analysis. Seven of the 16 orthologs in *V*. *chiguensis*, including *trkA2*, *smpB*, *nadA*, *mtnN2*, *rfbP*, *lon*, and *atpC*, exhibited expression profiles identical to those in *H*. *beimenensis*, suggesting that these 7 orthologous genes have identical functions in different phyla. Based on these high-throughput data, several halotolerance-related *V*. *chiguensis* genes were highlighted.

## Materials and methods

### Bacterial strain and growth conditions

*V*. *chiguensis* was incubated in growth medium [5 g/L yeast extract (Bacto, BD), 5 g/L casamino acids (Bacto, BD) and 5 g/L MgSO_4_·7H_2_O (Wako), pH 7.5] at 37°C with shaking at 220 rpm. For the halotolerant evaluation, the bacteria were incubated with various NaCl concentrations (0%, 5%, 10%, 15%, 20%, 25%) in the growth medium. To monitor the *V*. *chiguensis* concentration, a spectrophotometer (Libra S4, Biochrom) was used to measure three replicates per condition at OD_600_ every 6 h until 48 h.

### Genomic DNA and total RNA extraction and whole-transcriptome sequencing

For genomic DNA extraction, the bacteria were growth in 5% NaCl with growth medium for 24 h (OD_600_ = 1.0), and the Qiagen Gentra Puregene Kit (Qiagen) was used for DNA preparation. For transcriptome analysis, total RNA was extracted from bacterial cultures grown in 5% NaCl and 20% NaCl by a Total RNA Purification Kit (Geneaid). Genomic DNA and transcriptome sequencing were performed using Illumina MiSeq (2 × 300) paired-end sequencing by Genomics, BioSci & Tech Co.

### Genomic DNA assembly, gene prediction, annotation, gene comparison, and phylogenetic tree analysis

The draft genome sequence of *V*. *chiguensis* was *de novo* assembled by the Velvet Assembler v1.2.09 with default parameters [14]. For gene prediction, Rapid Annotation using Subsystem Technology (RAST; version 2.0) on the PARTIC platform (www.patricbrc.org/portal/portal/patric/Home) was used to annotate gene name and function [15, 16]. In addition, the genome GC content was predicted by PARTIC. tRNAscan [17] and RNAmmer [18] were used for transfer RNA (tRNAs) and ribosomal RNA (rRNA) prediction. BLAST2GO (version 3.3.5) with default settings was used for Gene Ontology (GO) analysis [19], and NCBI’s COG database (version 2014) (www.ncbi.nlm.nih.gov/COG/) [20] was used for clusters of orthologous groups of proteins (COG) analysis. Four *Virgibacillus* spp., including *V*. *halodenitrificans*, *V*. *necropolis*, *Virgibacillus* sp. SK37, and *Virgibacillus* sp. LM2416, were used for ortholog group clustering by OrthoMCL (orthomcl.org/orthomcl/) [21] to assess the gain and loss of genes in *V*. *chiguensis*. Furthermore, the ortholog groups from various phyla, such as *Bacillus subtilis* (CP021889), *H*. *beimenensis* (CP021435), *Escherichia coli* (NC_000913), and *Pseudomonas aeruginosa* (NC_002516), were compared with *V*. *chiguensis* to understand their similarities.

16S rRNA was used for phylogenetic tree analysis. The genus of *Virgibacillus* was compared by the neighbor-joining method with 1,000 bootstraps via MEGA v7 (Kumar et al., 2016). *Virgibacillus* spp. include *V*. *alimentarius* (GU202420.1), *V*. *campisalis* (GU586225.1), *V*. *halotolerans* (HE577174.1), *V*. *natechei* (JX435821.1), *V*. *byunsanensis* (FJ357159.1), *V*. *zhanjiangensis* (FJ425904.1), *V*. *carmonensis* (AJ316302.1), *V*. *necropolis* (AJ315056.1), *V*. *siamensis* (AB365482.1), *V*. *litoralis* (FJ425909.1), *V*. *salinus* (FM205010.1), *V*. *subterraneus* (FJ746573.1), *V*. *halophilus* (AB243851.1), *V*. *soli* (EU213011.1), *V*. *sediminis* (AY121430.1), *V*. *xinjiangensis* (DQ664543.1), *V*. *kekensis* (AY121439.1), *V*. *marismortui* (AJ009793.1), *V*. *salarius* (AB197851.2), *V*. *olivae* (DQ139839.3), *V*. *proomii* (AJ012667.1), *V*. *pantothenticus* (D16275.1), *V*. *dokdonensis* (AY822043.1), *V*. *chiguensis* strain NTU-101 (NR044086.1), *V*. *halodenitrificans* (AY543169.1), *V*. *albus* (JQ680032.1), and *V*. *koreensis* (AY616012.1). *Bacillus subtilis* (AJ276351.1) was selected as the outgroup in this phylogenetic tree.

### Transcriptome data processing and differential gene expression analysis

The transcriptome reads of the 5% and 20% NaCl samples were mapped to the genome draft sequence of *V*. *chiguensis* by Bowtie2 (version 2.2.5) [22] for gene expression analysis. The eXpress software (version 1.5) was used to calculate expression, which was presented as fragments per kilobase of transcript per million mapped reads (FPKM) values [23]. We set the absolute value of the log_2_ fold-change (log_2_FC) in FPKM as greater than 2 for differential expression. The transcriptome database of *V*. *chiguensis* was constructed in the ContigViews system (www.contigviews.bioagri.ntu.edu.tw).

### Quantitative reverse transcription-polymerase chain reaction (qRT-PCR)

qRT-PCR was performed to confirm the gene expressions of the transcriptome patterns of *V*. *chiguensis* in 5% and 20% NaCl. The primers that were used in this study were designed using the GenScript Real-time PCR Primer Design system (www.genscript.com/tools/real-time-pcr-tagman-primer-design-tool). The sequences of the primer sets are listed in S1 Table.

## Results and discussion

### Growth conditions and phylogenetic position of *V*. *chiguensis*

**Fig 1:**
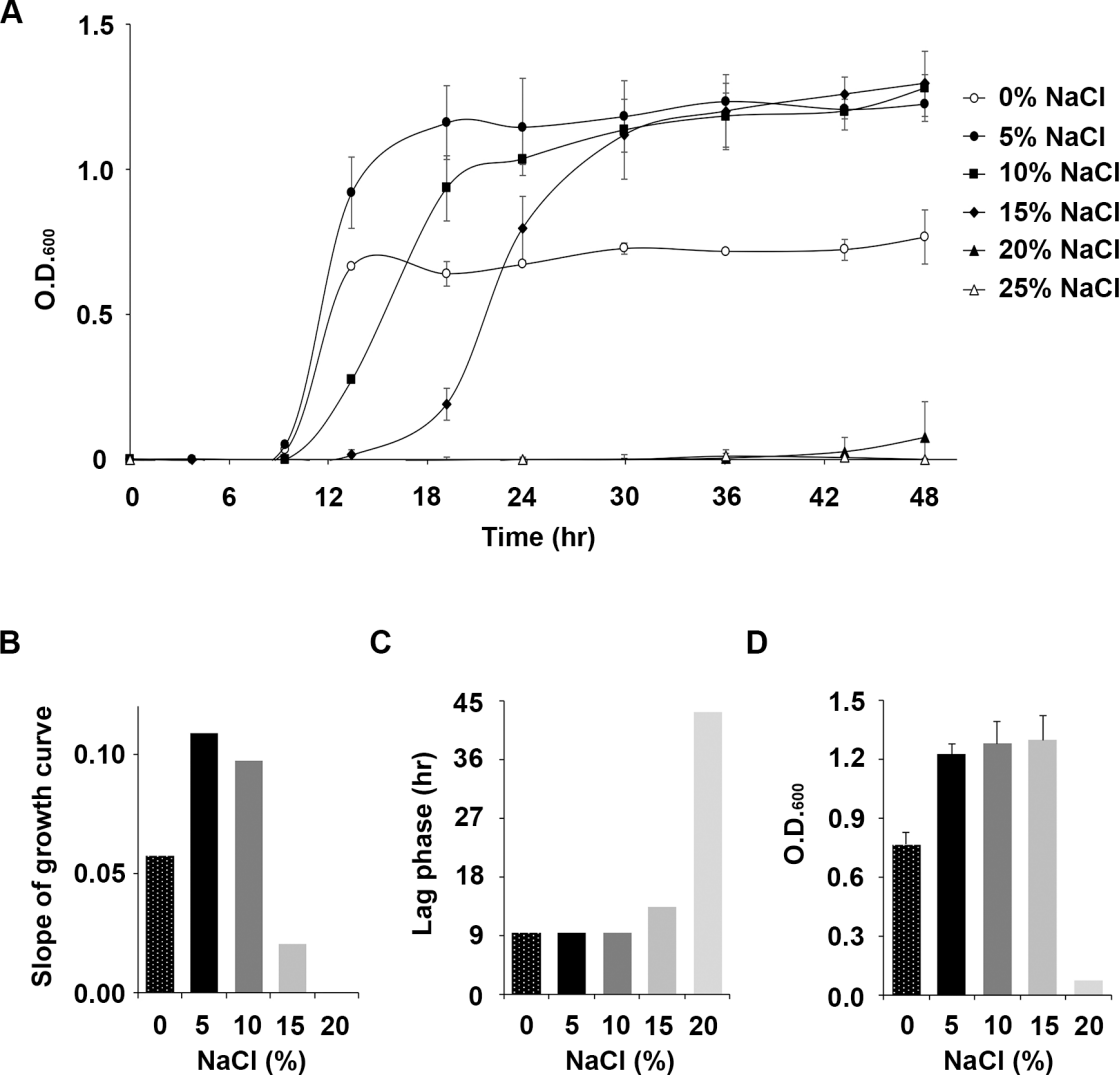
Growth conditions for *Virgibacillus chiguensis*. (A) Growth curves of *V*. *chiguensis* in 0%, 5%, 10%, 15%, 20%, and 25% (w/v) NaCl medium. (B) The slope of the growth curve of *V*. *chiguensis* between 9 and 19 h in the various NaCl concentrations. (C) The length of the lag phase for *V*. *chiguensis* growth under different NaCl conditions. (D) The OD_600_ concentration of *V*. *chiguensis* under various NaCl conditions at 48 h.

*V*. *chiguensis* could survive in concentrations of NaCl between 0% and 20% (Fig 1A). We found that *V*. *chiguensis* had a higher growth rate in 5% NaCl (0.11) than at the other NaCl concentrations between 9 h and 19 h, whereas no detectable growth was observed in 25% NaCl (Fig 1B). In addition, a lag phase of *V*. *chiguensis* in 0%, 5%, and 10% was observed at 9.4 h, whereas the phase in 15% and 20% conditions was later then 13.4 and 43.2 h, respectively (Fig 1C). *V*. *chiguensis* exhibited a fast growth rate in 5% NaCl, whereas the bacteria were inhibited at > 20% NaCl (Fig 1A and 1D). Moreover, there were no significant differences in maximal biomass under 5%, 10%, and 15% conditions at 48 h (Fig 1D). Thus, *V*. *chiguensis* can be classified as a moderately halophilic bacterium because it exhibits halotolerance at 10 to 20% NaCl and its optimal growth condition is 5% NaCl.

**Fig 2:**
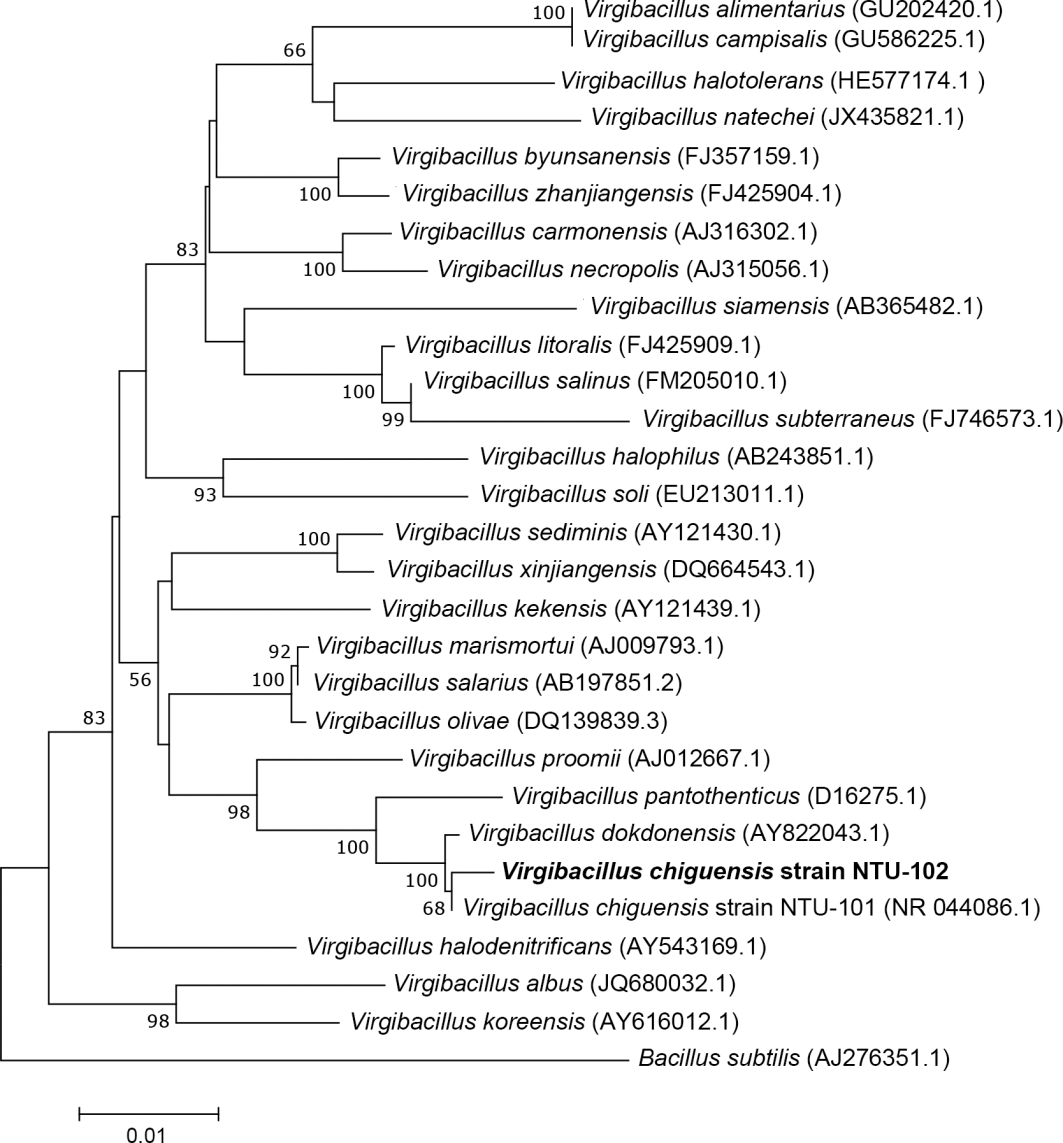
Phylogenetic tree for *Virgibacillus* spp. The 16S rRNA phylogenetic tree for *Virgibacillus* spp. was generated by the neighbor-joining method with Juke-Cantor correction. Bootstrap = 1,000. Bar, 0.01 substitutions per nucleotide position.

The phylogenetic tree of 16S rRNA showed that *V*. *chiguensis* was closely related to *V*. *chiguensis* strain NTU-101 and *V*. *dokdonensis*, with 99.5% identity (Fig 2). Notably, NTU-102 and NTU-101 were isolated from the same location.

### Genomic features of *V*. *chiguensis*

**Fig 3:**
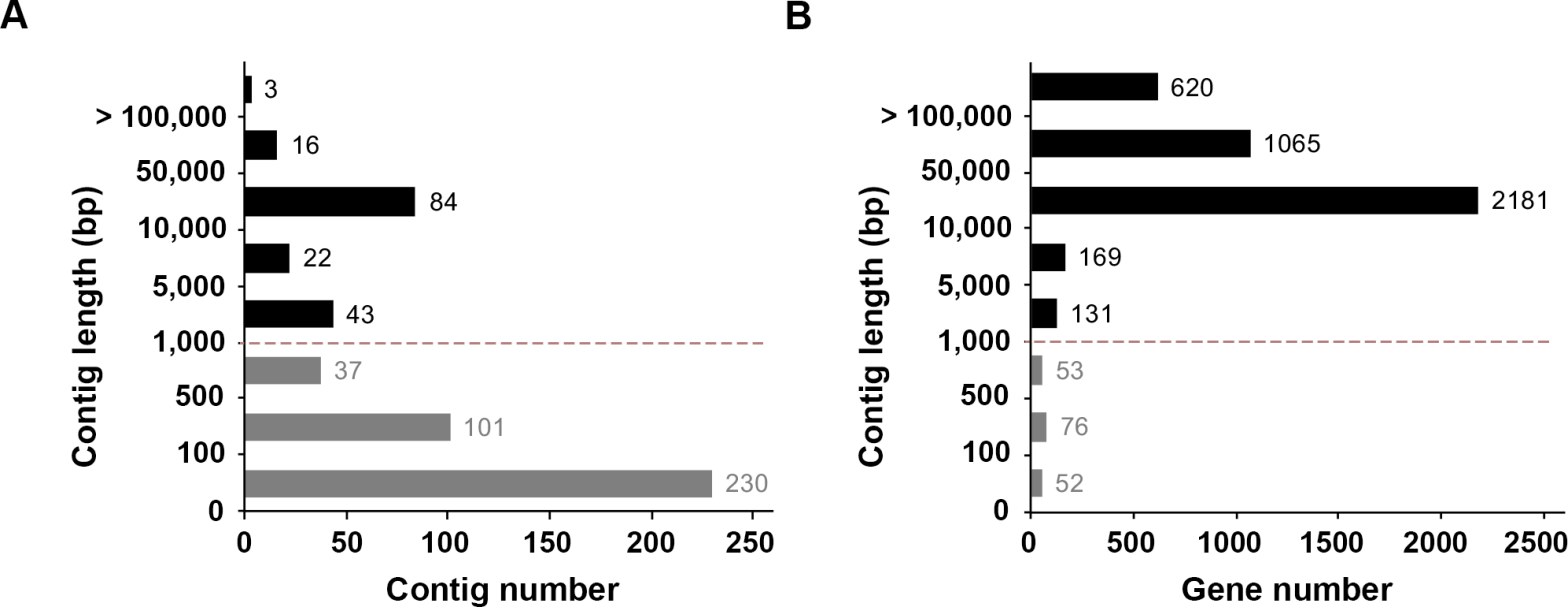
Contig length and gene number distributions of *Virgibacillus chiguensis*. (A) The contig length distribution of *Virgibacillus chiguensis*. The x-axis represents the number of contigs. The y-axis represents the contig length range. Bp, base pair. (B) The gene number distribution of *Virgibacillus chiguensis*. The x-axis represents the number of genes. The y-axis represents the contig length range. Bp, base pair.

The draft genomic contigs of *V*. *chiguensis* were *de novo* assembled by Velvet Assembler V1.2.09 with 3,719,926 reads. In total, we obtained 536 contigs of genomic DNA, with 103 contigs longer than 10,000 bp (Fig 3A). Sixty-five contigs were 1,000 to 10,000 bp, whereas the lengths of 37 contigs were 500 to 1,000 bp (Fig 3A). In addition, 101 contigs were 100 to 500 bp, and 230 contigs were smaller than 100 bp (Fig 3A). In general, 96% of genes were found on contigs longer than 1 kb (Fig 3B). In addition, 84 contigs were 10 kb– 50 kb in length, containing 2,181 genes (Fig 3A and 3B). We removed contigs shorter than 1,000 bp and used the remaining contig sequences (> 1 kb) to generate the draft genome of *V*. *chiguensis* (Fig 3A and 3B; S1 Dataset). The draft genome of *V*. *chiguensis* was 4,094,375 bp in length, and the largest contig was 272,869 bp. The N50-value was 42,585 bp. Furthermore, a total of 4,166 coding sequences (CDSs) were spread across the positive strand (2,136 CDSs) and negative strand (2,031 CDSs), and the genome was predicted to contain 3 rRNAs and 51 tRNAs. The average GC content was 36.58%. For comparison, the genomes size of the CGMCC 1.6496 strain is 4.13 Mb, with 3,787 CDS, similar to the values for *V*. *chiguensis* strain NTU-102, suggesting that our sequencing results are credible.

**Fig 4:**
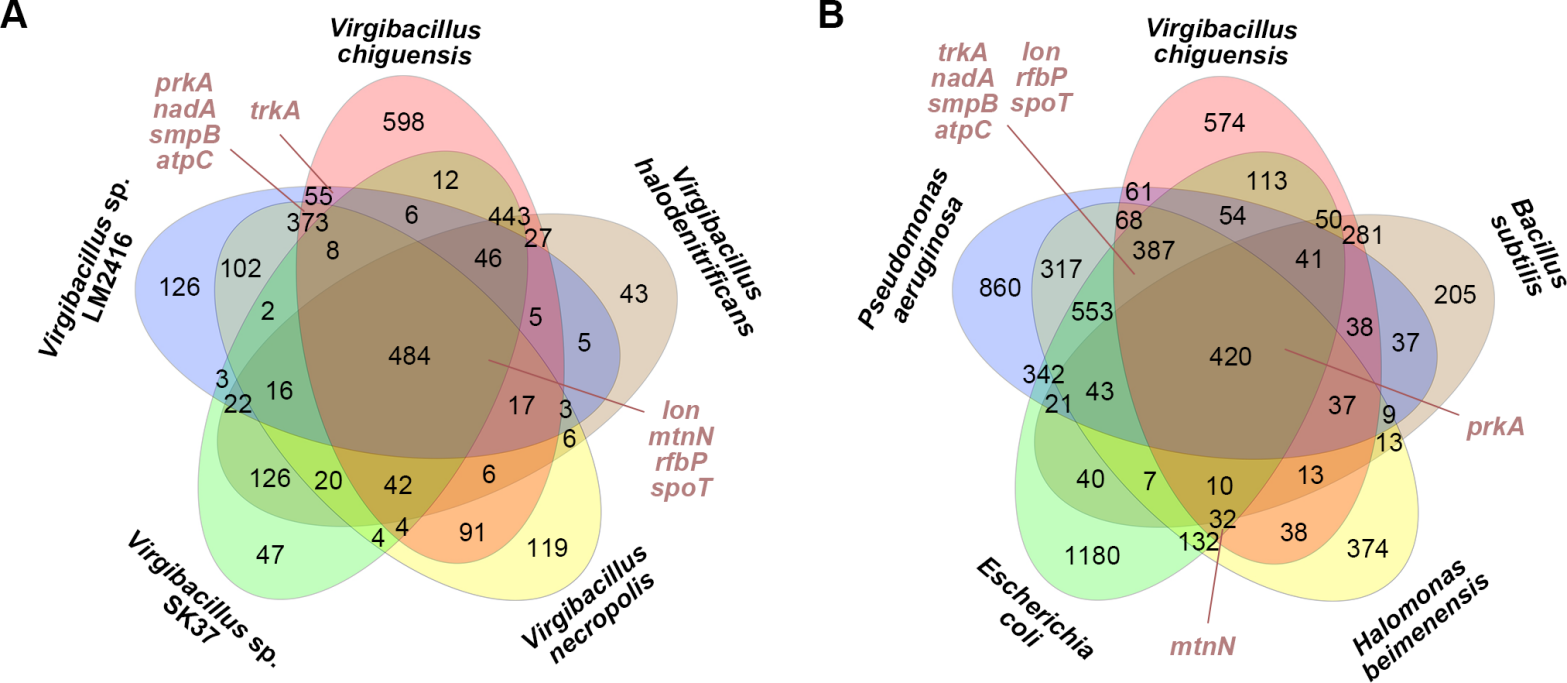
Gene comparison of *Virgibacillus chiguensis* with other species. (A) Venn diagram showing the gain and loss of genes between *V*. *chiguensis* and four *Virgibacillus* spp.: *V*. *halodenitrificans*, *V*. *necropolis*, *Virgibacillus* sp. SK37, and *Virgibacillus* sp. LM2416. (B) Venn diagram showing the gain and loss of genes between *V*. *chiguensis* and four other species, including *Halomonas beimenensis* (CP021435), *Escherichia coli* (NC_000913), *Pseudomonas aeruginosa* (NC_002516), and *Bacillus subtilis* (CP021889). The distributions of the 9 orthologous halotolerant-related genes in *V*. *chiguensis* and *H*. *beimenensis* are indicated on the Venn diagrams.

A total of 484 orthologous genes were detected in *Virgibacillus* spp., including orthologs of genes from *V*. *halodenitrificans*, *V*. *necropolis*, *Virgibacillus* sp. SK37, and *Virgibacillus* sp. LM2416 (Fig 4A). *V*. *chiguensis* contains 598 unique genes and lacks 644 genes that are present in the 4 other *Virgibacillus* spp. (Fig 4A). A total of 1,643 genes are shared between *V*. *chiguensis* and either *H*. *beimenensis*, *E*. *coli*, *P*. *aeruginosa*, or *B*. *subtilis*. By contrast, there are 574 unique genes in *V*. *chiguensis* (Fig 4B).

### GO and COG analysis

**Fig 5:**
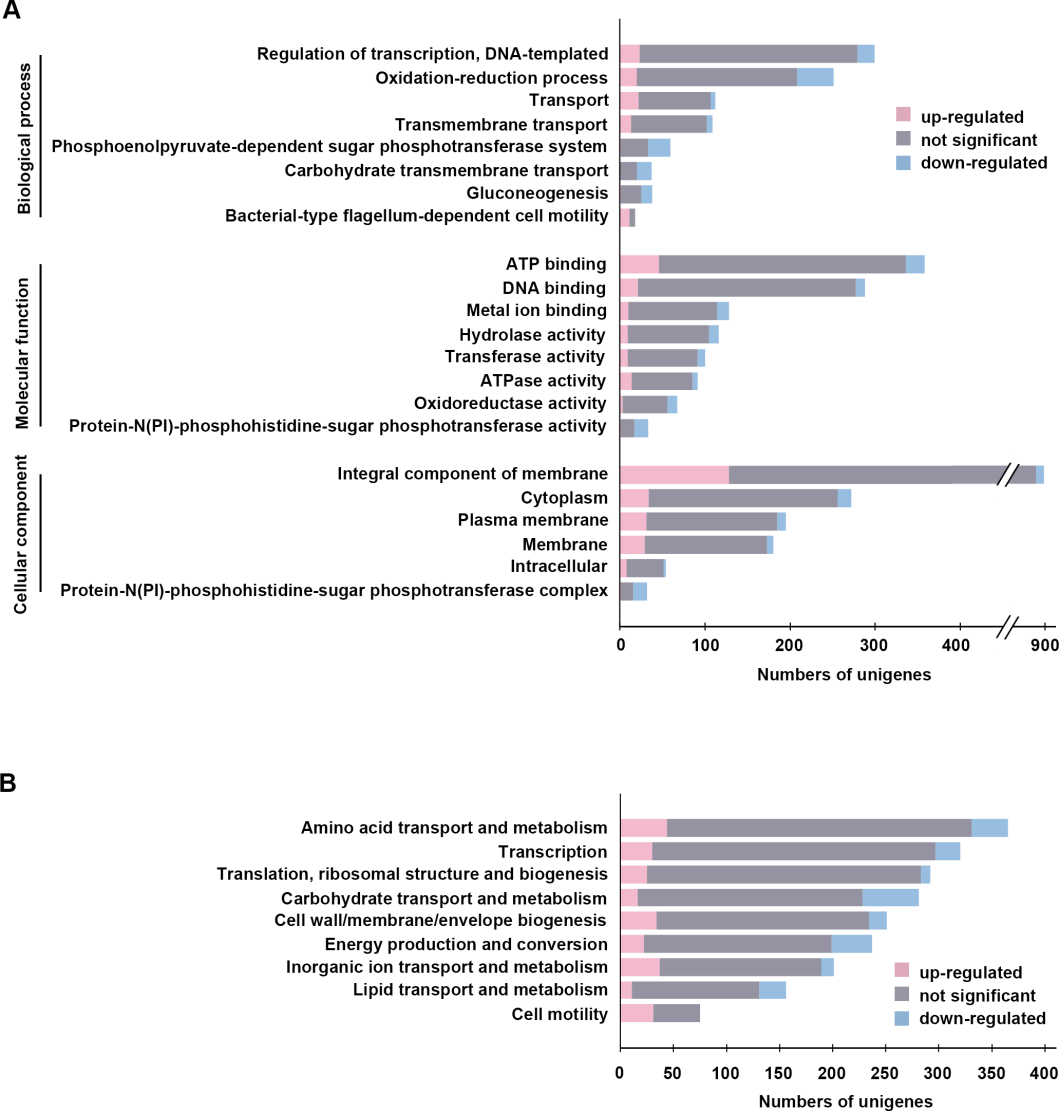
Gene features of *Virgibacillus chiguensis* for gene ontology (GO) and clusters of orthologous groups of proteins (COG). (A) The top 5 GO sub-categories for DEGs of *V*. *chiguensis*. The pink bars represent up-regulated DEGs (log_2_FC of FPKM > 2); the blue bars represent down-regulated DEGs (log_2_FC of FPKM < −2); the gray bars represent DEGs that did not significantly change. (B) The top 5 clusters of COG categories for the DEGs of *V*. *chiguensis*.

Based on the gene annotation, 3,037 genes with 10,567 hits in the NCBI nr database (each gene may be assigned more than one GO term) were assigned to three GO categories (Fig 5A). In the “cellular components” category, there were 103 sub-categories with 2,123 hits (Fig 5A); in the “molecular function” category, there were 829 sub-categories with 3,939 hits (Fig 5A); and in the “biological process” category, there were 685 sub-categories with 4,504 hits (Fig 5A). The genes in the cellular components category were assigned to sub-categories that included “integral component of membrane” (873 genes), “cytoplasm” (272 genes), and “plasma membrane” (195 genes) (Fig 5A). The genes in the molecular functions category were assigned to sub-categories that included “ATP binding” (358 genes), “DNA binding” (288 genes), and “metal ion binding” (128 genes) (Fig 5A). Moreover, the genes in the biological processes category were assigned to sub-categories that included “regulation of transcription, DNA-templated” (299 genes), “oxidation-reduction process” (251 genes), and “transport” (112 genes) (Fig 5A). The details of the GO sub-categories and gene information are listed in S2 Dataset.

Based on the sequence homology, a total of 4,043 genes were mapped to 22 different COG categories (Fig 5B). The top three mapped COG categories were “Amino acid transport and metabolism” (365 genes), “Transcription” (320 genes), and “Translation, ribosomal structure, and biogenesis” (292 genes). Overall, these 3 categories accounted for 24.17% (977 of 4,043 genes) of the annotated COG functional dataset (Fig 5B). The details of the COG sub-categories and gene information are listed in S2 Dataset.

### Transcriptome profiles of *V*. *chiguensis*

The transcriptome profiles of *V*. *chiguensis* grown in 5% and 20% NaCl were used to identify differentially expressed genes (DEGs). The transcriptome reads obtained from bacteria grown in 5% (7,493,634 reads) and 20% (7,507,500 reads) NaCl were compared to the genomic sequence to determine the mapping rate and to perform FPKM calculations. Our data indicated that approximately 95.49% (5% NaCl) and 96.94% (20% NaCl) of the transcriptome reads were mapped to the draft genomic sequence. The transcriptome data are available on the ContigViews system [24].

**Fig 6:**
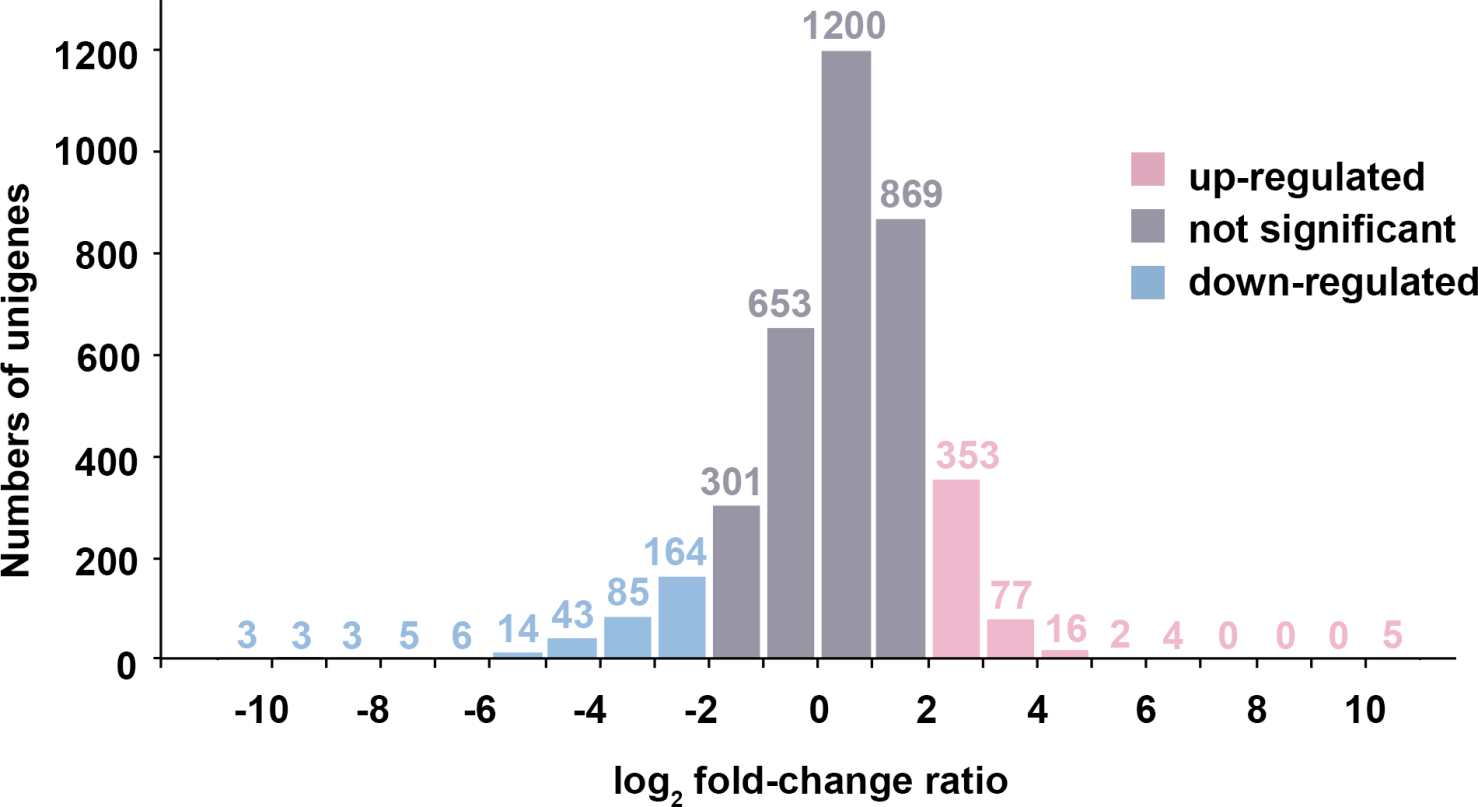
Differentially expressed genes (DEGs) of *Virgibacillus chiguensis* among various NaCl conditions. The up-regulated DEGs are represented by pink columns showing log_2_FC of FPKM was > 2; the down-regulated DEGs are represented by blue columns showing log_2_FC of FPKM was < -2; the not significantly changed genes are represented by gray columns showing log_2_FC of FPKM did not exceed 2. The numbers indicate the number of genes.

We identified 783 DEGs (457 up-regulated genes and 326 down-regulated genes) showing a log_2_FC in FPKM > 2 between the 5% and 20% NaCl conditions (Fig 6). The top GO and COG categories, with the corresponding number of DEGs, are highlighted in Fig 5.

The top three GO cellular component sub-categories of the genes up-regulated in 20% NaCl were “integral component of membrane” (128 genes), “cytoplasm” (34 genes), and “plasma membrane” (31 genes), whereas the top three sub-categories of the genes down-regulated in 20% NaCl were “integral component of membrane” (48 genes), “cytoplasm” (16 genes), and “plasma membrane” (10 genes) (Fig 5A). The top three GO molecular function sub-categories of the genes up-regulated in 20% NaCl were “ATP binding” (46 genes), “DNA binding” (21 genes), and “ATPase activity” (14 genes), whereas the top three sub-categories of the genes down-regulated in 20% NaCl were “ATP binding” (22 genes), “protein-N(PI)-phosphohistidine-sugar phosphotransferase activity” (16 genes), and “metal ion binding” (14 genes) (Fig 5A). Moreover, the top three GO biological process sub-categories of the genes up-regulated in 20% NaCl were “DNA-templated regulation of transcription” (23 genes), “transport” (22 genes), and “oxidation-reduction process” (20 genes), whereas the top three sub-categories of the genes down-regulated in 20% NaCl were “oxidation-reduction process” (43 genes), “phosphoenolpyruvate-dependent sugar phosphotransferase system phosphotransferase activity” (26 genes), and “DNA-templated regulation of transcription” (20 genes) (Fig 5B). The details of the GO sub-categories and gene information are listed in S2 Dataset.

The up-regulated genes were classified into 19 COG functional categories (S2 Dataset), the top five of which were “amino acid transport and metabolism” (44 genes), “inorganic ion transport and metabolism” (37 genes), “cell wall/membrane/envelope biogenesis” (34 genes), “cell motility” (31 genes), and “transcription” (30 genes) (Fig 5B). In addition, the genes that were down-regulated in 20% NaCl were classified into 18 COG functional categories (S2 Dataset). The top five were “carbohydrate transport and metabolism” (53 genes), “energy production and conversion” (38 genes), “amino acid transport and metabolism” (34 genes), “lipid transport and metabolism” (25 genes), and “transcription” (23 genes) (Fig 5B). The details of the COG sub-categories and gene information are listed in S2 Dataset.

### Expression profiles of halotolerant-related genes in *V*. *chiguensis*

Our previous study of *H*. *beimenensis* identified 16 genes involved in halotolerance [13]. Here, we searched for orthologous *V*. *chiguensis* genes and identified 9 orthologs, including the potassium transporter gene *trkA2*, tmRNA-binding protein *smpB*, quinolinate synthetase gene *nadA*, 5'-methylthioadenosine/S-adenosylhomocysteine nucleosidase gene *mtnN2*, undecaprenyl-phosphate galactose phosphotransferase gene *rfbP*, (p)ppGpp synthetase/guanosine-3',5'-bis(diphosphate) 3'-diphosphatase gene *spoT*, ATP-dependent protease gene *lon*, PrkA family serine protein kinase gene *prkA*, and ATP synthase gene *atpC* (S1 File).

Orthologs of genes such as *trkA2*, *atpC*, and *nadA* have significant biological functions in halotolerance, controlling potassium uptake, hydrogen ion transport for energy conversion, and compatible solute synthesis [13]. Other genes such as *spoT*, *prkA*, *mtnN2*, *lon*, *smpB*, and *rfbP* also have critical functions in promoting halotolerance, including cellular signaling, quorum sensing, translation, and cell motility.

The orthologous genes, such as *lon*, *mtnN2*, *rfbP*, and *spoT*, were blasted in all five *Virgibacillus* species (Fig 4A). Genes including *prkA*, *nadA*, *smpB*, and *atpC* were blasted in *V*. *chiguensis*, *V*. *necropolis*, and *Virgibacillus* sp. LM2416 (Fig 4A). The ortholog *trkA2* was only blasted in *V*. *chiguensis* and *Virgibacillus* sp. LM2416 (Fig 4A). Moreover, in different phyla, the ortholog *prkA* was blasted in *V*. *chiguensis*, *B*. *subtilis*, *H*. *beimenensis*, *E*. *coli*, and *P*. *aeruginosa* (Fig 4B). Seven orthologous genes, i.e., *lon*, *rfbP*, *nadA*, *smpB*, *atpC*, *spoT*, and *trkA2*, were blasted in *V*. *chiguensis*, *H*. *beimenensis*, *E*. *coli*, and *P*. *aeruginosa* (Fig 4B). However, the ortholog *mtnN2* was blasted in *V*. *chiguensis*, *H*. *beimenensis*, and *E*. *coli* (Fig 4B). These results indicate that some of halotolerant-related genes are conserved in different species and even in different phyla. Notably, the cutoff value for blasting against OrthoMCL proteins is e^-5^ and 50% match. If the best OrthoMCL protein is below the threshold, the protein will not be assigned to any ortholog group from the database.

**Fig 7:**
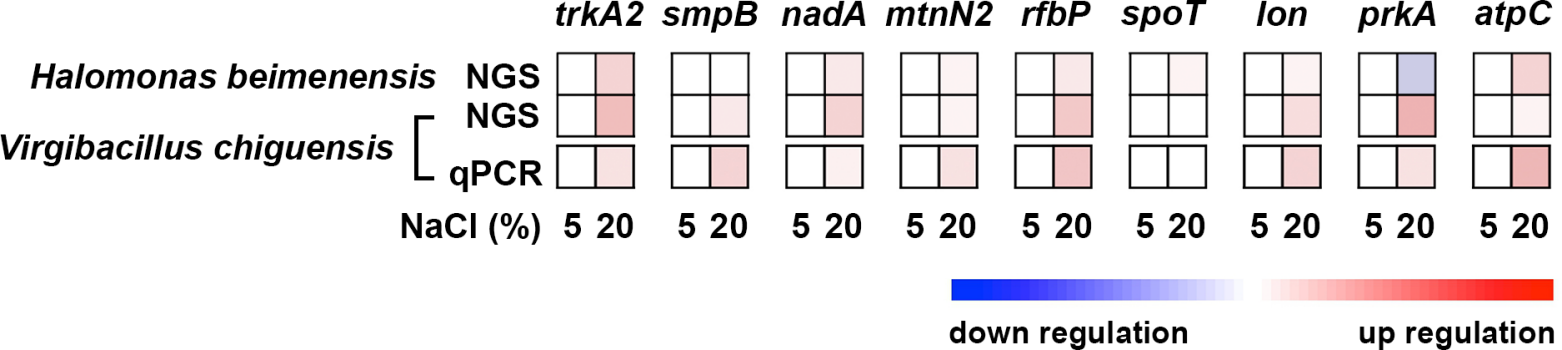
Gene expression of halotolerance-related genes in *Halomonas beimenensis* and *Virgibacillus chiguensis* based on the transcriptome profiles and qRT-PCR validation. Genes related to halotolerance included the potassium transporter gene (*trkA2*), tmRNA-binding protein (*smpB*), quinolinate synthetase gene (*nadA*), 5'-methylthioadenosine/S-adenosylhomocysteine nucleosidase gene (*mtnN2*), undecaprenyl-phosphate galactosephosphotransferase gene (*rfbP*), (p)ppGpp synthetase/guanosine-3',5'-bis(diphosphate) 3'-diphosphatase gene (*spoT*), ATP-dependent protease gene (*lon*), PrkA family serine protein kinase gene (*prkA*), and ATP synthase gene (*atpC*).

The transcriptome profile showed that 7 of these 9 *V*. *chiguensis* genes were up-regulated in 20% NaCl (Fig 7). Compared to the expression profiles in *H*. *beimenensis*, the gene expression profiles in *V*. *chiguensis* were quite similar, except for those of *prkA* and *spoT* (Fig 7). Notably, the gene expression of *spoT* in *V*.*chiguensis* did not differ significantly between the two conditions (Fig 7). In *V*. *chiguensis*, the expression of *prkA* was up-regulated, whereas it was down-regulated in *H*. *beimenensis* (Fig 7). These data strongly suggest that 7 of the 9 identified orthologous genes, including *trkA2*, *smpB*, *nadA*, *mtnN2*, *rfbP*, *lon*, and *atpC*, are involved in halotolerance in a different phylum. The gene expression of these 9 genes was validated by qRT-PCR, and the results were agreed with the NGS transcriptome patterns (Fig 7).

In *H*. *beimenensis*, *rfbP* and *mtnN2* mutants affect swarming ability [13]. *rfbP* encodes an undecaprenyl-phosphate galactose phosphotransferase [13], whereas *mtnN* of *V*. *cholera* encodes a 5'-methythioadenosine nucleosidase for the quorum-sensing pathway [13]. The *atpC* and *trkA2* genes encode subunit epsilon of ATP synthase and are involves in potassium uptake, respectively, which are related to energy production [13]. SmpB forms a tmRNP complex to monitor trans-translation [13]. NadA is involved in compatible solute biosynthesis [13], whereas Lon plays roles in homeostasis for halotolerance [13]. These genes are critical for the halotolerance of *H*. *beimenensis* [13]. Therefore, these 7 genes may also be used as marker genes or indicators for screening halotolerant species.

## Conclusion

Our draft genome and transcriptome profiles results demonstrated that omics data are helpful in exploring saline adaptation mechanisms and could be used to identify potential halotolerance-related enzymes in *V*. *chiguensis* in the future. Moreover, the data indicated that the profiles of the 7 halotolerant genes under high-saline conditions might be similar in *V*. *chiguensis* and *H*. *beimenensis*. Therefore, we propose that the 7 halotolerant marker genes identified in *H*. *beimenensis* could be used to screen new halotolerant species.

## Supporting information

S1 Dataset The sequences of the *Virgibacillus chiguensis* contigs. (TXT)

S2 Dataset Gene annotation of *Virgibacillus chiguensis* and gene expression profiles under different NaCl concentrations. (XLSX)

S1 File Nine figures of the alignment results of 9 identified orthologous genes that are involved in halotolerance in *V*. *chiguensis* and *H*. *beimenensis*. (PDF)

S1 Table Primer sets of *Virgibacillus chiguensis* for qRT-PCR. (PDF)
