# Supplementary material for: Characterization of candidate genes involved in halotolerance using high-throughput omics in the halotolerant bacterium Virgibacillus chiguensis
Source: PLoS One. 2018 Aug 9;13(8):e0201346. doi: 10.1371/journal.pone.0201346 (PMC6084883; doi:10.1371/journal.pone.0201346)
Supplement: S1 File — (PDF) [file pone.0201346.s003.pdf]

S1 File. Nine figures of the alignment results of 9 identified orthologous genes which involved in halotolerance in *V. chiguensis* (VC) and *H. beimenensis* (HB).

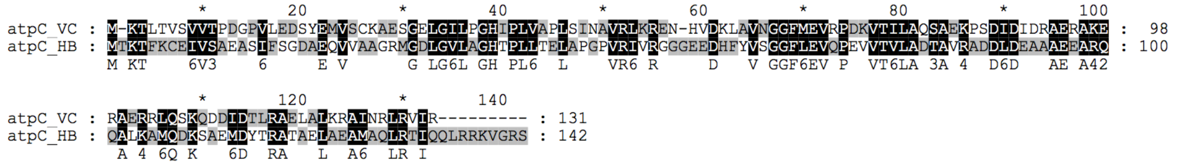

S1 Fig. The alignment result of *atpC* orthologous genes.

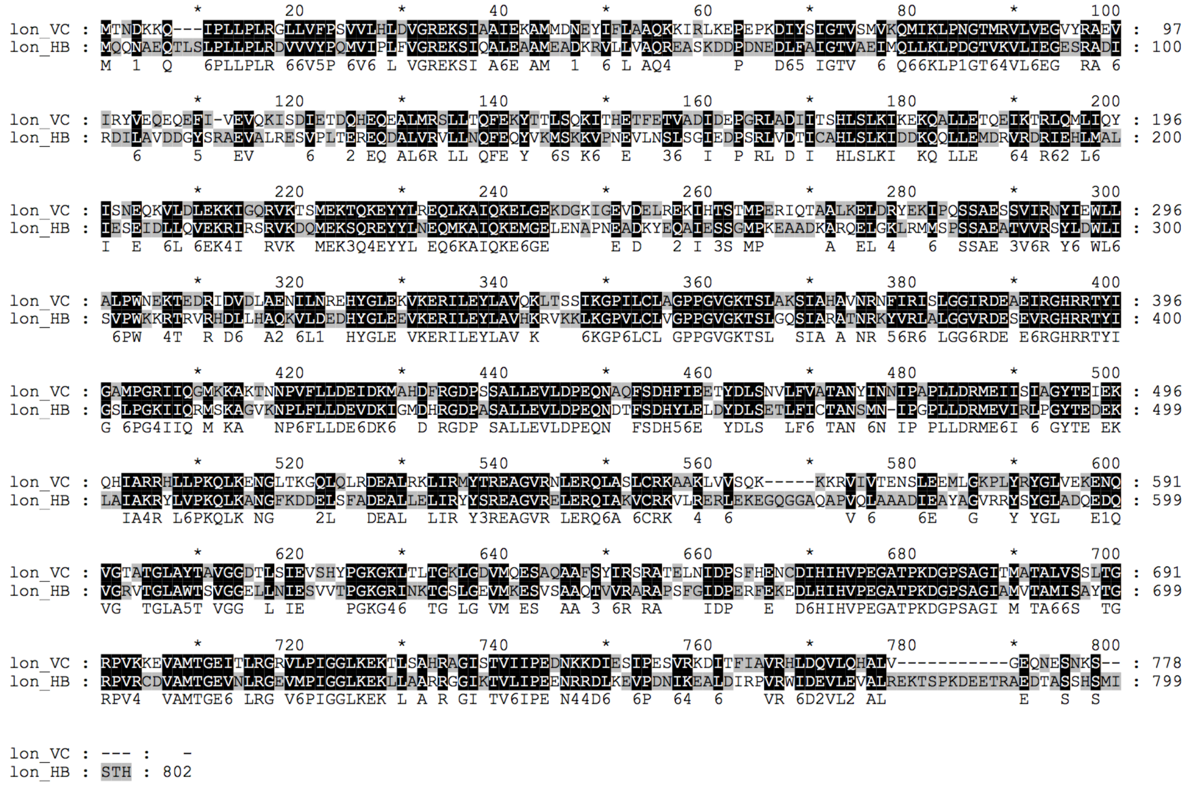

S2 Fig. The alignment result of *lon* orthologous genes.

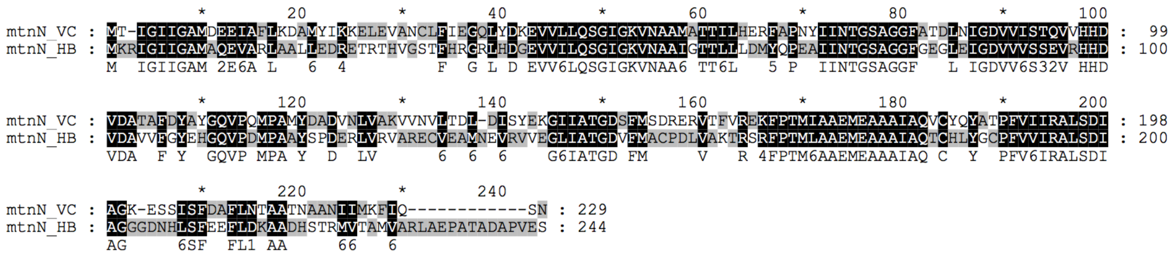

S3 Fig. The alignment result of *mtnN2* orthologous genes.

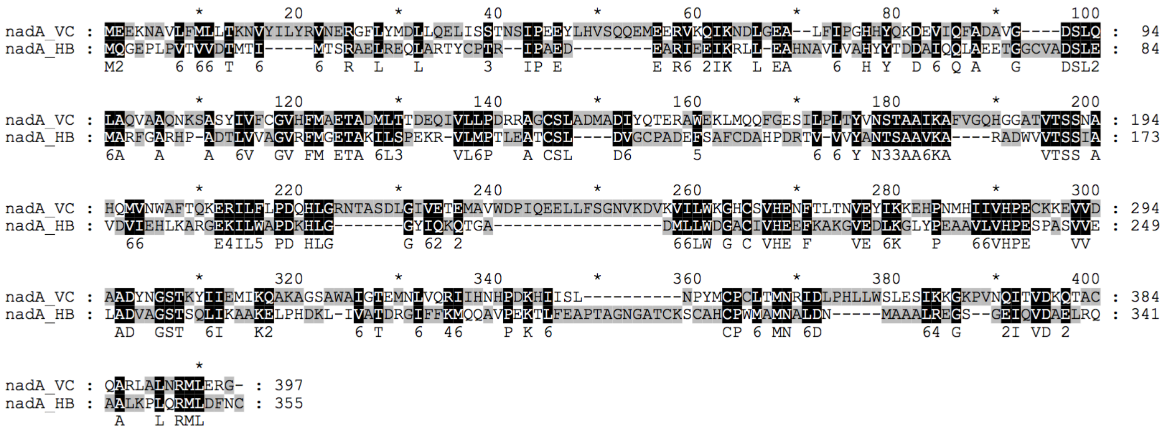

S4 Fig. The alignment result of *nadA* orthologous genes.

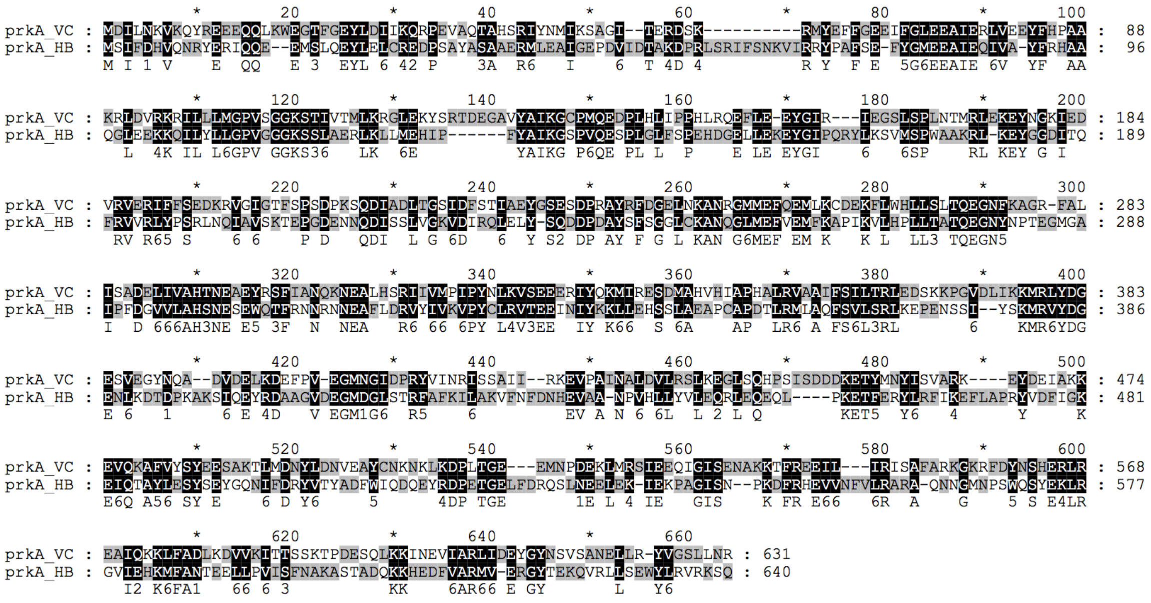

S5 Fig. The alignment result of *prkA* orthologous genes.

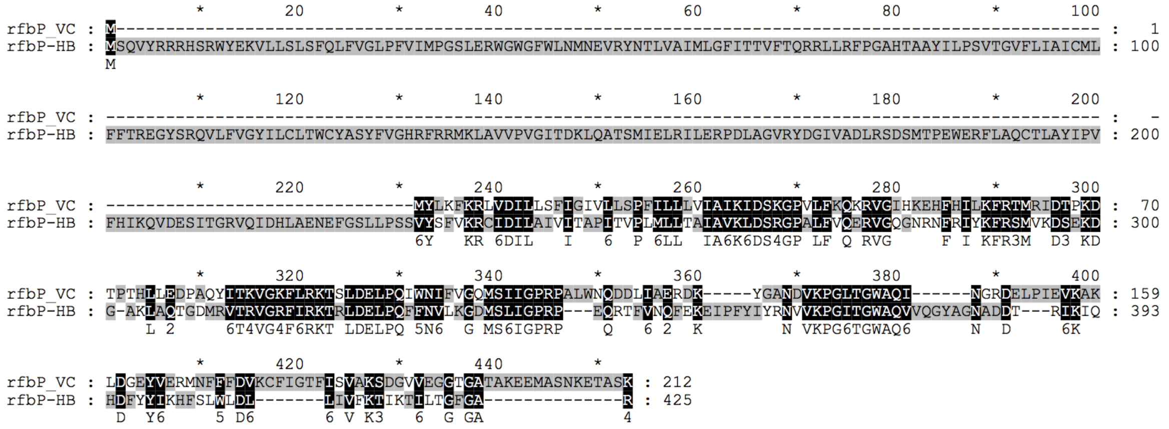

S6 Fig. The alignment result of *rfbP* orthologous genes.

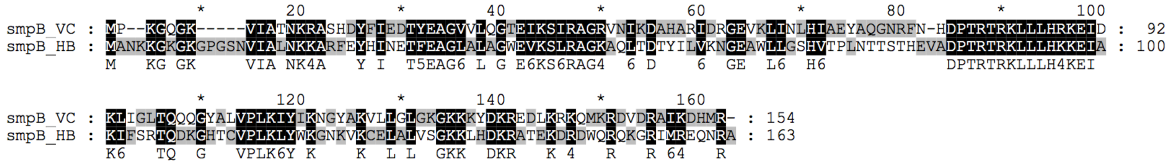

S7 Fig. The alignment result of *smpB* orthologous genes.

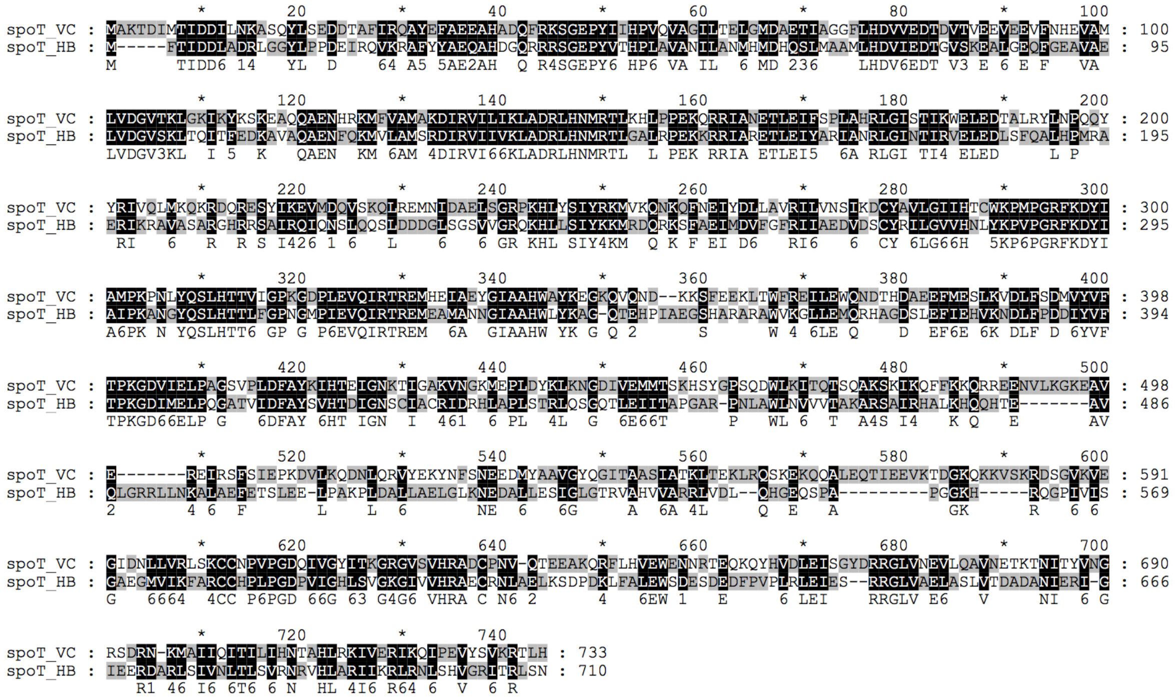

S8 Fig. The alignment result of *spoT* orthologous genes.

```
                 *        20         *        40         *        60         *        80         *       100
trkA_VC : MSYEMIVVAVVVCGMIGTLIMDIARPVIIVFTALTILLVTGILKPEDALAGFSNEGMLTIALLFVIAGAMQKSGMIEVIMKGWLNKSNRFIGAMIRVLIP : 100
trkA_HB : MSPDAWISLVVVTAVFPVMALTRIGPDMVLMGALVLLLGLGVVDPQQALGGFSSSGLFTVAFMYVLVASIRETGGIDLIIRYVLGRPKGERLALARLVLP : 100
          MS    6  VVV  6   6 6    P 666  AL 6LL  G66 P2 AL GFS  G6 T6A 65V6   6  3G I 6I64  L 4       A6 R666P

                 *       120         *       140         *       160         *       180         *       200
trkA_VC : TSALSAFFNNTPIVVTFTPMLKNWCEEHQIAPSKFLIPLSYATILGGTITLMGTSTNLVVHGMLID-YGYDGFSLFQLAVIGIPITVVGLIYLIVIGRHL : 199
trkA_HB : VAPLSGFLNNTPVVATFIPAVLSWSRRLNLPAHRLLMPLSFASILGGTITLLGTSTNLVVHGLLIERYPSLAMGLFDIAWVGVPVAVVGVTYLLLVGRRL : 200
             LS F NNTP6V TF P 6  W     6   4 L6PLS5A3ILGGTITL6GTSTNLVVHG6LI  Y      LF 6A 6G6P6 VVG6 YL666GR L

                 *       220         *       240         *       260         *       280         *       300
trkA_VC : LPDQKGFQQQVRIGAKEYMAELIVDHSFPGINQTVKEARLKELTGIYLLGIVRNKERVFPVRSTTRIQAGDRLIFTGLITALTGVQVTKGLTLS-MGKPI : 298
trkA_HB : LPPRQG-ATEVFENPREFTIEMEVDPAGPLVDRTVEEAGLRHLQELFLVEIERDGNVVSVVGPGERLKGGDRLVFAGTSAGAVELQQIRGLVPSHHGESS : 299
          LP   G  2V    4E5  E6 VD    P 61 TV EA L4 L  65L6 I R1   V  V    R6 GDRL6F G        6Q  4GL  S  G

                 *       320         *       340         *       360         *       380         *       400
trkA_VC : DVAELKKANSTIVEAVVSHQSSLVYQSIKQVKFRSLYDAGVIAVHRNNEQLQSKIGDILLKPGDHLLLLAGADFIDKYQQSEDFYVMMPFHRMHMVHRPS : 398
trkA_HB : LQKDFKERR--LVEAVVSDQCQFIGRRIRDGHFRTLYGAAVLAVCRGGERVAGNLGQIRLQPADVLLLEARPPFIERHRQSRDFLLIS---QLNGSARPA : 394
               K     6VEAVVS Q   6    I4    FR3LY A V6AV R  E 6   6G I L P D LLL A    FI 4  QS DF 66       6    RP

                 *       420         *       440         *       460         *       480         *       500
trkA_VC : F-KGWLTILIFLIMIILITIGVLSMFKAMAIVVAVMLAMKVVTMEEIKKYIHFDVLVLIACSFGIGIATMKSGLAPWIAD---GIVTVGKPLGLFTLLFL : 494
trkA_HB : HERAWLAWSILAGVVLLATTGLTSLLNAAMLGAAASVLSGCCTIGAAKRGLDTQVLLTLAASFGLGAALDASGAASALAGTAMGMVD-GNP---WWLLIG : 490
            4 WL   I   666L T G6 S6  A  6  A  6      T6   K4 6   VL6 6A SFG6G A   SG A  6A     G6V  G P    5 LL

                 *       520         *       540         *       560         *       580         *
trkA_VC : IYIVTNIFTEIITNSAAAILMIPIAFEMAETLGLDPMGFAVTVTIAASASFITPIGYQTNLIVYGPGSYTFKDYVKIGTPLSILVMFITV-IIVYFWWF : 592
trkA_HB : TYLVVALLTELVTNNAAAVITFPVVIAGAESLGVSPMPYVVAVMFAASASFLTPIGYQTNLMVYGPGGYRVSDYLRVGAPLNLLTAVVALGLIPIVWPF : 589
           Y6V  6 TE66TN AAA66  P6    AE3LG6 PM 5 V V  AASASF6TPIGYQTNL6VYGPG Y   DY646G PL 6L   6 6 6I    W F
```

S9 Fig. The alignment result of *trkA2* orthologous genes.
