# Supplementary material for: Characterization of candidate genes involved in halotolerance using high-throughput omics in the halotolerant bacterium Virgibacillus chiguensis
Source: PLoS One. 2018 Aug 9;13(8):e0201346. doi: 10.1371/journal.pone.0201346 (PMC6084883; doi:10.1371/journal.pone.0201346)
Supplement: S1 Table — (PDF) [file pone.0201346.s004.pdf]

S1 Table. Primer sets of *Virgibacillus chiguensis* for qRT-PCR

**Primer Name**	**Sequence (5′ to 3′)**
VCH_16S601	GGAGGGCCATTGGAAACTGG
VCH_16S704	CTTCGCCACTGGTGTTCCTC
VCH_atpC221	AAGTGACGATTTTAGCGCAGT
VCH_atpC325	CTTGTTTTGATTGGAGACGGC
VCH_lon881	GGTTGTTAGCGTTACCTTGGA
VCH_lon974	TCCAGACCGTAATGTTCCCT
VCH_mtnN359	ATGCAGATGTCAACTTGGTCG
VCH_mtnN455	ATAAACGAATCACCAGTCGCA
VCH_nadA659	CGGCTTCTGATCTCGGGATTG
VCH_nadA787	CGTGTACAGAGCAATGTCCCT
VCH_prkA260	CAGCGAAACGGCTCGATGTA
VCH_prkA396	CGCATAAACCGCTCCCTCAT
VCH_rfbP482	ACGGCGAATATGTAGAACGCA
VCH_rfbP615	GGAGGCCATTTCTTCCTTCGC
VCH_smpB111	GTCGATTCGTGCTGGAAGGG
VCH_smpB253	GCAGCTTTCTTGTACGAGTTGGA
VCH_spoT102	TGCCGAAGAAGCTCATGCTG
VCH_spoT216	TGCGATTGTTTCAGCGTCCA
VCH_trkA467	CGAATCTCGTTGTGCATGGC
VCH_trkA617	ACCCTTTCTGATCAGGCAACA
